# A descriptive cohort study of withdrawal from inhaled corticosteroids in COPD patients

**DOI:** 10.1038/s41533-022-00288-6

**Published:** 2022-07-20

**Authors:** Smit Patel, Scott Dickinson, Kevin Morris, Helen F. Ashdown, James D. Chalmers

**Affiliations:** 1 Boehringer Ingelheim Ltd, Bracknell, UK; 2 Nuffield Department of Primary Care Health Sciences, University of Oxford, Radcliffe Observatory Quarter, Oxford, UK; 3 Scottish Centre for Respiratory Research, University of Dundee, Ninewells Hospital and Medical School, Dundee, UK

**Keywords:** Chronic obstructive pulmonary disease, Health occupations, Epidemiology, Respiratory signs and symptoms, Outcomes research

## Abstract

Inhaled corticosteroid (ICS) therapy is widely prescribed without a history of exacerbations and consensus guidelines suggest withdrawal of ICS in these patients would reduce the risk of side effects and promote cost-effective prescribing. The study describes the prescribing behaviour in the United Kingdom (UK) in relation to ICS withdrawal and identifies clinical outcomes following withdrawal using primary and secondary care electronic health records between January 2012 and December 2017. Patients with a history ≥12 months’ exposure who withdrew ICS for ≥6 months were identified into two cohorts; those prescribed a long-acting bronchodilator maintenance therapy and those that were not prescribed any maintenance therapy. The duration of withdrawal, predictors of restarting ICS, and clinical outcomes were compared between both patient cohorts. Among 76,808 patients that had ≥1 prescription of ICS in the study period, 11,093 patients (14%) withdrew ICS therapy at least once during the study period. The median time without ICS was 9 months (IQR 7–14), with the majority (71%) receiving subsequent ICS prescriptions after withdrawal. Patients receiving maintenance therapy with a COPD review at withdrawal were 28% less likely to restart ICS (HR: 0.72, 95% CI 0.61, 0.85). Overall, 69% and 89% of patients that withdrew ICS had no recorded exacerbation event or COPD hospitalisation, respectively, during the withdrawal. This study provides evidence that most patients withdrawing from ICS do not experience COPD exacerbations and withdrawal success can be achieved by carefully planning routine COPD reviews whilst optimising the use of available maintenance therapies.

## Introduction

International clinical guidelines on the management of Chronic Obstructive Pulmonary Disease (COPD), Global Initiative for Chronic Obstructive Lung Disease (GOLD) 2020^1–3^ recommend long-acting inhaled bronchodilators, including β2-agonists (LABA) and anti-muscarinic agents (LAMA) as maintenance therapies. These agents can be prescribed as a monotherapy dose, fixed dose of dual LAMA/LABA therapy, or in combination with inhaled corticosteroids (ICS) for the symptomatic management of COPD and the prevention of COPD-related exacerbations.

The GOLD treatment strategy recommends that ICS (prescribed as a combination inhaler with a LABA or as part of triple-therapy regimen with LAMA and LABA) are reserved for COPD patients with frequent or severe exacerbations, and research shows that certain features might identify patients more or less likely to respond well to treatment with ICS, including asthma-like phenotype or biomarkers such as eosinophilia^4–8^. However, considerable dissociation has been reported between guideline recommendations and clinicians’ practices^9–11^ despite significant efforts to promote and disseminate the GOLD strategy^12^. ICS are often inappropriately prescribed for patients with mild or moderate COPD without a history of exacerbations resulting in up to 70% of patients in current practice receiving ICS-containing regimens^13,14^. There is a lack of evidence of the benefit of ICS in patients with preserved lung function and no history of exacerbations^15,16^. Safety concerns, particularly regarding increased incidence of pneumonia, osteoporosis, bruising, adrenal suppression, and diabetes have been reported in ICS users^17^.

It has been proposed that ICS should be withdrawn in patients who may have been prescribed this therapy inappropriately, to reduce side effects and promote cost-effective prescribing^18–20^. The recently published European Respiratory Society (ERS) guidelines on ICS withdrawal suggest patients withdrawing ICS should receive ongoing maintenance therapy with a bronchodilator^21^. While guidelines and clinical opinion have been shared around how to withdraw ICS^22–25^, there is limited evidence on the extent to which withdrawal recommendations have been adopted in primary care^26^. The aim of this study is to describe the trends in ICS withdrawal and the outcomes for patients receiving long-acting bronchodilator maintenance therapy (LAMA or LABA monotherapy or combination dual therapy), compared to patients not receiving any maintenance bronchodilators. Secondly, this study will aim to identify patient characteristics associated with successful withdrawal of ICS.

## Methods

### Study design and Inclusion criteria

This retrospective cohort study included patients belonging to UK general practices contributing data to the Clinical Practice Research Datalink (CPRD), the world’s largest longitudinal primary care database that is utilised for pharmacoepidemiologic research^27,28^. The data encompasses approximately 60 million patients; GPs voluntarily contribute the data which is automatically transferred from the routine patient care and it is representative of the UK population^27^.

Included patients in this study were those with a confirmed COPD record for adults ≥35 years of age at diagnosis, where the diagnosis of a COPD was defined using any record of a diagnostic Read Code for COPD in primary care records or COPD ICD-10 code in hospital records. The accuracy of COPD diagnosis in CPRD has been previously validated^29^. Patients were included if they were withdrawing a long-term ICS prescription between January 2012 to December 2017. To identify a group of patients with long-term ICS prescription, we required a minimum historical period of 12 months’ persistent exposure to ICS therapy (alone or in any combination) after allowing for the therapy period (calculated using amount prescribed or an imputed 28 days where data were missing). Additionally, patients were required to be currently registered in the observational period (January 2012 to December 2017) in general practices that contain research quality data linked with patient and practice-level secondary care hospital data in the Hospital Episode Statistics (HES) database. The final sample size for the study was based on the above basic eligibility criteria in addition to ICS-withdrawal requirements.

### Definition of ICS withdrawal and data capture

Since gaps in prescription records of a few months are common within CPRD, a minimum ICS-free period was required to identify true withdrawal rather than a short period during which patients did not receive prescriptions. For the purposes of analysis, we therefore defined “withdrawal” as a period of at least 6 months with no record of ICS prescription. Patients’ withdrawal period was then considered from the index date—defined as the last date of ICS therapy based on the duration of medication prescribed, until the earlier date of subsequent ICS prescription (alone or in any combination), study end, acceptable research quality data (patient remains registered in GP practice without any data gaps), transferring out of the registered practice, or death. For patients with multiple periods of ICS withdrawal, we considered the first occurring period. Data capture consisted of a 2-year period prior to ICS withdrawal and minimum 6 months period after the withdrawal date to identify clinical outcomes.

### Outcomes of interest

Outcomes of interest in this study included time without ICS; the number of exacerbations (defined as antibiotic and oral corticosteroid (OCS) prescriptions taken together for 5–14 days or lower respiratory tract infection Read Code, or an acute exacerbation Read Code where that did not coincide with a spirometry test)^30^ during the patient’s ICS-withdrawal period; the number of COPD hospitalisations (ICD-10 code J44.0, J44.1 or J22 in any position of hospital episode); the number of interactions with a general practitioner (GP); the number of short-acting bronchodilator prescriptions (i.e. rescue therapy); and number of pneumonia episodes (Read Code and ICD-10 codes J12-J16 and J18). Measurements of these outcomes were taken from distinct patient groups (patients receiving bronchodilation maintenance therapy compared to patients not receiving bronchodilation therapy).

### Patient characteristics and covariates

Baseline characteristics included age at the start of withdrawal, sex, BMI, smoking status, vaccination history (influenza or pneumococcal vaccination), co-morbidities (heart disease or asthma), prior history of COPD exacerbations, recent eosinophil count and percent predicted forced expiratory volume in 1 s (FEV1). In addition, the coding of routine COPD reviews (6 or 12-month intervals) with spirometry procedure on the day was also recorded. All clinical and demographic covariates were collected at baseline using the primary care electronic health data in CPRD.

Two distinct patient groups were defined—those receiving one or more long-acting bronchodilator maintenance therapies during the ICS-withdrawal period, and those with no long-acting bronchodilator treatment during the period of ICS withdrawal. Long-acting bronchodilator treatment in the maintenance group consisted of LAMA or LABA monotherapy, LAMA/LABA combination fixed dose, or LAMA + LABA combination free dose which includes separate inhalers prescribed for intended regular daily usage, not for as-needed use^31^.

### Statistical analysis

Baseline characteristics and follow-up patient outcomes were described for patients receiving or not receiving inhaled maintenance therapy during their ICS-withdrawal period. Cross-tabulated summary statistics were used to describe these patient characteristics and included mean ± standard deviation (SD) for continuous data and number (percentage) for categorical data. Missing data for covariates were quantified without imputation due to the relative percentage of missing data.

Kaplan–Meier plots to describe the probability of ICS-free time (months) and median ICS-free time (months) were calculated in patients that were prescribed a maintenance therapy compared with those without maintenance therapies. The frequency and percentage of patients lost to follow-up were also quantified by corresponding reasons for the loss to follow-up. A univariable Cox-proportional hazards model was initially performed to identify significant predictors of restarting ICS therapy at the level of *α* = 0.05. These significant predictors were used to develop a two-sided multivariable predictive model using backwards variable selection at the level of *α* = 0.2 to produce a parsimonious complete-case model adjusting for confounders. Hazard function (hazard ratio) for patients on maintenance therapy and for patients without maintenance therapy after withdrawal are assumed to be proportional, with a constant hazard ratio over time. Potential interactions were also tested using the final multivariable model. For these analyses, those who received their first maintenance therapy prescription after the first 6 months of withdrawal were considered not on maintenance therapy at the index to avoid immortal time bias.

In a sensitivity analysis, we used a time-dependent variable, which changed from no maintenance therapy to the use of maintenance therapy in patients who received their first maintenance therapy after the first 6 months. In an additional sensitivity analysis, the median duration of ICS-free time was repeated based on a minimum 9 months of ICS-free time prior to the index date to understand whether a longer baseline ICS-free time would influence the number of exacerbations in patients receiving maintenance treatment or not receiving maintenance treatment.

The analyses were performed in accordance with relevant regulations and guidelines. This study was reviewed and approved by the Independent Scientific Advisory Committee for Medicines and Healthcare products Regulatory Agency (MHRA) database research (ISAC number 17195RA) and by an internal scientific committee of the study sponsor. As this was a non-interventional study using anonymised data, no patient consent was necessary. Primary and secondary database access and data extraction were restricted to the corresponding author who was also responsible for the creation of the final study population and statistical analysis. Raw data extracts were assessed for outliers and cleaned to provide a clinically and statistically complete dataset. Data linkage between primary and secondary care data was performed by merging and appending based on a common patient identification number for both databases. Covariates were stratified by clinical thresholds and aggregated for *n* < 10 patients. All cleaning methods and analyses were conducted using SAS (SAS Institute Inc., Cary, NC, USA) and STATA/IC 15.0.

### Reporting summary

Further information on research design is available in the Nature Research Reporting Summary linked to this article.

## Results

### Cohort identification and characteristics

**Fig. 1:**
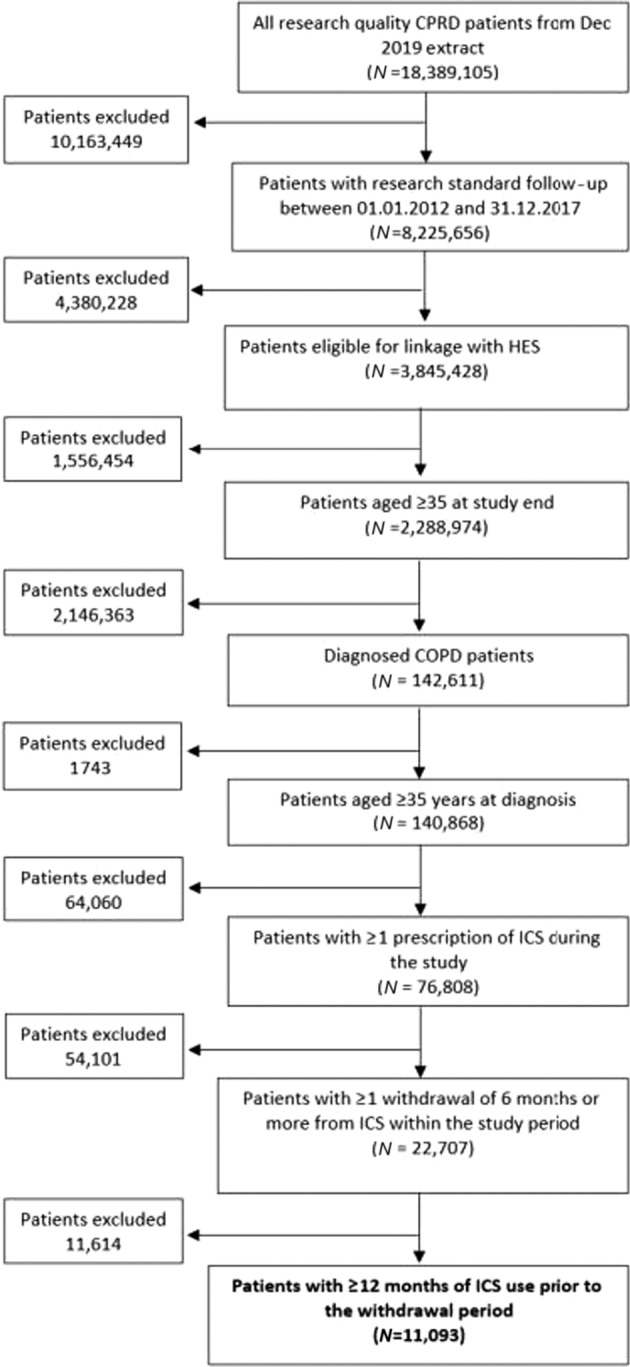
Inclusion and exclusion of COPD study population in the UK. The number of patients that are eligible and included in the study population is illustrated in the flow chart.

We identified 76,808 COPD patients that had ≥1 prescription of ICS in the study period. Of these patients, 11,093 (14.4%) withdrew an ICS therapy at least once during the study period having had >12 months use of ICS prior to this withdrawal (Fig. 1). Overall, these patients had a mean age of 70 (SD 15.6) and 52.8% were female. During the observed withdrawal period, 3849 patients (34.7%) were prescribed ≥1 maintenance therapy and 7244 patients (65.3%) were not prescribed any maintenance therapy. Of those who were prescribed maintenance therapy, 343 (8.9%) received their first prescription after the initial 6-month window that defined withdrawal. Patients who were prescribed a maintenance therapy during the withdrawal period were more likely to have FEV_1_% of <50% (maintenance therapy: 22.7%; no maintenance therapy: 10.7%; *P* < 0.001), MRC dyspnoea score of ≥3 (maintenance therapy: 47.5%; no maintenance therapy: 21.5%; *P* < 0.001) and be classified as GOLD group D (maintenance therapy: 20%; no maintenance therapy: 7.9%; *P* < 0.001). In addition, patients on maintenance therapy had higher hospitalised or non-hospitalised exacerbations (maintenance therapy: 59.3%; no maintenance therapy: 41.3%) and a higher percentage of pneumonia diagnosis (maintenance therapy: 23.2%; no maintenance therapy: 17.2%; *P* < 0.001) in the year prior to the withdrawal of ICS (Table 1).

**Table 1:** Characteristics of patients withdrawing ICS.

Characteristic^a^	All patients (*N* = 11,093)		Patients receiving maintenance therapy (*N* = 3849)		No maintenance therapy prescribed (*N* = 7244)	
Female	5857	52.8%	1896	49.3%	3961	54.7%
Mean ± SD age, years	70.0	15.6	71.5	11.0	69.1	13.3
35–44	351	3.2%	44	1.1%	307	4.2%
45–54	1101	9.9%	256	6.7%	845	11.7%
55–64	2019	18.2%	666	17.3%	1353	18.7%
65–74	3221	29%	1239	32.2%	1982	27.4%
75–80	1905	17.2%	762	19.8%	1143	15.8%
>80	2496	22.5%	882	22.9%	1614	22.3%
Mean ± SD body mass index (BMI) kg/m^2^	28.1	6.6	28.0	6.6	28.2	6.6
Missing^b^	2914	26.3%	431	11.2%	2483	34.3%
Smoking status						
Current smoker	2920	26.3%	1115	29%	1805	24.9%
Non-smoker/never	1025	9.2%	236	6.1%	789	10.9%
Ex-smoker	5349	48.2%	2330	60.5%	3019	41.7%
Data not entered	1799	16.2%	168	4.4%	1631	22.5%
Mean ± SD years since COPD diagnosis	5.1	6.3	6.8	6.2	4.3	6.2
Missing^b^	484	4.4%	180	4.7%	304	4.2%
Mean ± SD latest stable blood eosinophil count (absolute count × 10^9^ cells/L)	0.23	0.15	0.23	0.15	0.23	0.15
Missing^b^	2718	24.5%	888	23.1%	1830	25.3%
Mean ± SD latest FEV1 % of predicted	65%	21.2%	61%	20.4%	67%	21.4%
GOLD 1— ≥80	1561	14.1%	517	13.4%	1044	14.4%
GOLD 2—50–79	3250	29.3%	1538	40.0%	1712	23.6%
GOLD 3—30–49	1358	12.2%	724	18.8%	634	8.8%
GOLD 4—<30	587	5.3%	152	3.9%	135	1.9%
Missing^b^	4637	41.8%	918	23.9%	3719	51.3%
MRC Dyspnoea Score						
1–2	3328	30.0%	1334	34.7%	1994	27.5%
≥3	3382	30.5%	1827	47.5%	1555	21.5%
Missing^b^	4383	39.5%	688	17.9%	3695	51.0%
CAT Score						
<10	149	1.3%	80	2.1%	69	1.0%
≥10	394	3.6%	216	5.6%	178	2.5%
Missing^b^	10,550	95.1%	3553	92.3%	6997	96.6%
Exacerbations (mean ± SD in year prior)	1.0	1.8	1.4	2.1	0.8	1.5
Hospitalised exacerbation	964	8.7%	424	11.0%	540	7.5%
Non-hospitalised exacerbation	4311	38.9%	1859	48.3%	2452	33.8%
GOLD Group (2020)						
A	2720	23.6%	1032	26.8%	1688	23.3%
B	2055	17.8%	1072	27.9%	983	13.6%
C	609	5.3%	303	7.9%	306	4.2%
D	1329	11.5%	755	20%	574	7.9%
Undetermined	4380	38.0%	687	17.8%	3693	51.0%
Asthma history						
>2 years before COPD diagnosis	4665	42.1%	1320	34.3%	3345	46.2%
≤2 years before COPD diagnosis or withdrawal date	3394	30.6%	1251	32.5%	2413	33.3%
No history	3034	27.4%	1278	33.2%	1486	20.5%
Pneumonia (year prior)	2136	19.3%	892	23.2%	1244	17.2%

^a^*N*,% unless otherwise stated.

^b^Imputation methods were not conducted due to the high amount of missing data for stable blood eosinophil count, FEV1% GOLD groups, MRC dyspnoea scores, and CAT scores.

### Withdrawal period characteristics

Among patients prescribed maintenance therapy during the ICS-withdrawal period, 77% (*n* = 2965 of 3849) received a LAMA monotherapy. Despite lower utilisation of dual therapy compared with monotherapy, patients receiving LAMA/LABA fixed-dose or LAMA + LABA free-dose combination were observed to remain on ICS-free therapy on average longer than patients taking monotherapy (LAMA/LABA: 18.3 months, LAMA + LABA: 20.2 months; LAMA: 13.6 months, LABA: 14.7 months), respectively. Of all patients that withdrew with maintenance therapy, 22.8% (*n* = 877 of 3849) had a COPD review recorded which coincided at the start of their withdrawal period and 10.5% (*n* = 364 of 3849) at the end of withdrawal. Similarly, patients without maintenance therapy prescribed were more likely to have a COPD review at the start of withdrawal (7.1%, *n* = 517 of 7244) than at the end (4.5%, *n* = 326 of 7244). Withdrawal period ended due to a subsequent prescription of an ICS therapy (70.7%; *n* = 7846 of 11,093) compared with patients that were lost to follow-up which included patients records that were no longer of research quality (14.1%, *n* = 1567 of 11,093), transferred out of a GP (4.5%, *n* = 497 of 11,093), or died (4%; *n* = 446 of 11,093). In this study, 6.7%, 739 of 11,093 patients reached the study end date without restarting ICS (Supplementary Table 1).

Patients with a subsequent ICS prescription were likely to commence the same drug and device as the last ICS therapy prior to withdrawal (83%; *n* = 6515 of 7846). Although patients were overall less likely to be prescribed a different ICS therapy at the end of withdrawal, patients receiving maintenance therapy were more likely to switch to a different ICS therapy (25%, *n* = 627 of 2521) than those without maintenance therapy (13.2%; *n* = 704 of 5325) (Table 2).

**Table 2:** Characteristics of withdrawal periods in patients withdrawal ICS.

Characteristic^a^	All patients (*N* = 11,093)		Patients receiving maintenance therapy (*N* = 3849)^b^		No maintenance therapy prescribed (*N* = 7244)	
Maintenance therapy prescribed^b^						
LAMA + LABA^c^	~	~	265	6.9%	~	~
Mean months withdrawn ICS (95% CI)	~	~	20.2	18.4, 22	~	~
LAMA/LABA^d^	~	~	276	7.2%	~	~
Mean months withdrawn ICS (95% CI)	~	~	18.3	16.9, 19.8	~	~
LAMA only	~	~	2,965	77%	~	~
Mean months withdrawn ICS (95% CI)	~	~	13.6	13.2, 14.1	~	~
LABA only	~	~	345	9%	~	~
Mean months withdrawn ICS (95% CI)	~	~	14.7	13.3, 16	~	~
Withdrawal periods that end due to ICS prescription	7846	70.7%	2521	65.5%	5325	73.5%
Last ICS therapy is the same as the first	6515	83.0%	1894	75.1%	4621	86.8%
ICS therapy is different	1331	17.0%	627	24.9%	704	13.2%
Withdrawal periods coinciding with a COPD annual/6-monthly review	1929	17.4%	1132	29.4%	797	11.0%
At start of withdrawal	1394	12.6%	877	22.8%	517	7.1%
At end of withdrawal	690	6.2%	364	9.5%	326	4.5%

^a^*N*,% unless otherwise stated.

^b^Included patients observed to have clear periods of using different maintenance therapy regimens.

^c^Free-dose combination.

^d^Fixed-dose combination.

**Fig. 2:**
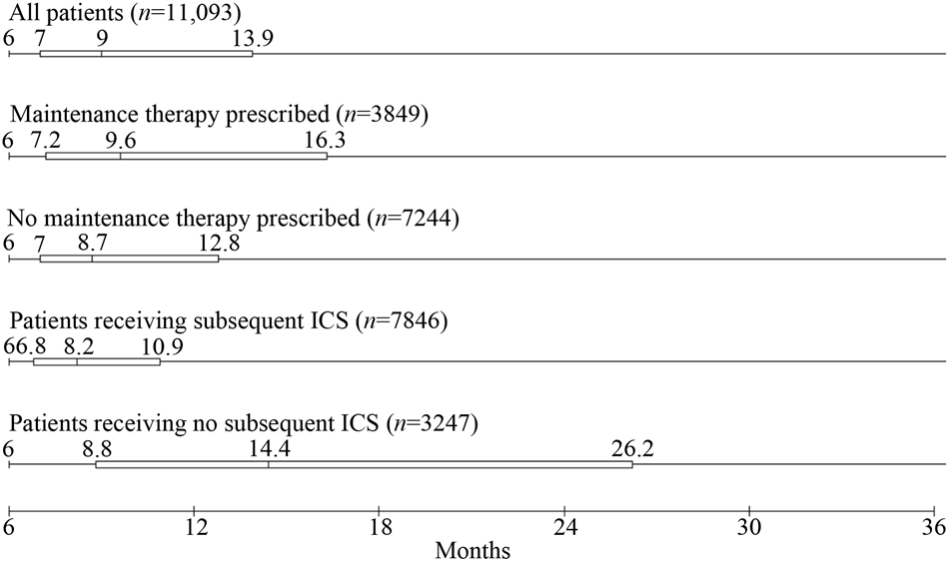
Distribution of observed ICS-free follow-up time among COPD patients by patient group. The duration of withdrawal for patients receiving or not receiving maintenance therapy during withdrawal and or ICS after withdrawal is illustrated in this figure. Data shown represent the minimum, lower quartile, median, and upper quartile. Maximum values not shown (maximum = 76 months among patients receiving a subsequent prescription of ICS; maximum = 77 months in all other groups).

**Fig. 3:**
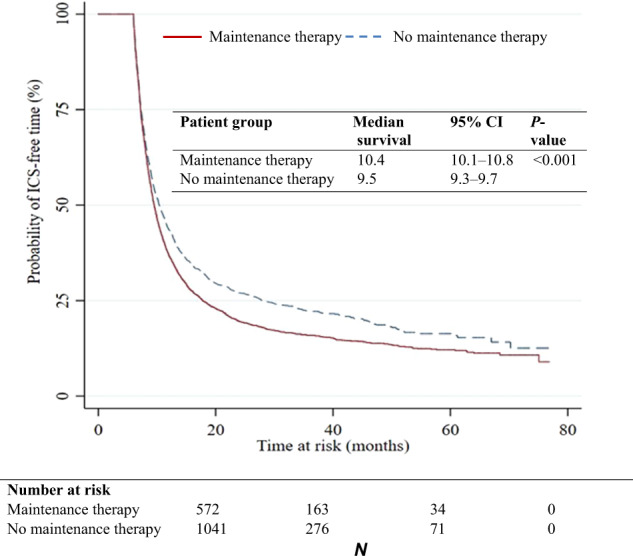
Kaplan–Meier analysis for time without ICS among COPD patients withdrawing therapy by the patient group. The median duration of ICS-withdrawal time is illustrated based on the time at risk for patients on maintenance therapy and patients without maintenance therapy.

The observed median ICS-free time was 9 months (IQR 7–13.9). This differed slightly when comparing whether patients were prescribed a maintenance therapy during the withdrawal period or not (median: 9.6 months vs. 8.7 months, respectively) (Fig. 2). After accounting for censoring, the estimated median time without ICS in patients withdrawing with maintenance therapy and without maintenance therapy was 10.4 months (95% CI: 10.1, 10.8) and 9.5 months (95% CI: 9.3, 9.7), respectively as illustrated in the Kaplan–Meier curve (Fig. 3). The mean withdrawal period was consistently higher in patients receiving maintenance therapy in each calendar year compared with patients not receiving maintenance therapy (Supplementary Fig. 1).

### Characteristics associated with time without ICS

The Cox-proportional hazards model presented in Table 3 illustrates the predictors associated with ICS-free time. In the univariable analysis, the use of maintenance therapy, a COPD review at start of withdrawal of ICS, absence of heart disease, and higher FEV1% predicted were each more likely to be associated with a longer time without ICS. Patients with an asthma history, increasing blood eosinophil count, prior exacerbations, and vaccination history were associated with a shorter time without ICS. The multivariable model illustrated that patient with asthma history of >2 years before COPD diagnosis and ≤2 years before COPD diagnosis or withdrawal were 28% (HR: 1.28; 95% CI: 1.18, 1.38; *P* < 0.001) and 12% (HR: 1.12; 95% CI: 1.04, 1.22; *P* < 0.001) more likely to restart ICS compared with patients without a history of asthma, respectively. Although statistically significant (*P* < 0.001), FEV1% and exacerbation history were marginally associated with ICS-free time (Table 3).

**Table 3:** Univariable and multivariable model of time without ICS based on patient characteristics (hazard ratios >1 present increased risk of restarting ICS or shorter-ICS free time).

	Univariable analysis		Multivariable analysis^a^ (*N* = 6009)	
Characteristic	HR (95% CI)	*P* value	HR (95% CI)	*P* value
Age				
35–44	Reference		Reference	
45–54	0.94 (0.82, 1.08)	0.38	0.96 (0.74, 1.25)	0.75
55–64	0.95 (0.84, 1.08)	0.46	0.98 (0.77, 1.26)	0.88
65–74	0.91 (0.80, 1.03)	0.14	0.97 (0.75, 1.24)	0.80
75–80	0.90 (0.79, 1.02)	0.10	0.97 (0.75, 1.25)	0.82
>80	0.83 (0.73, 0.94)	0.003	0.94 (0.73–1.21)	0.63
Sex				
Female	Reference		Reference	
Male	0.96 (0.92, 1.0)	0.064	0.95 (0.89, 1.01)	0.12
Body mass index (BMI) kg/m^2^				
Normal	Reference		Reference	
Underweight	1.13 (0.95, 1.35)	0.16	0.98 (0.79, 1.21)	0.84
Overweight	1.11 (0.96, 1.28)	0.17	1.02 (0.85, 1.22)	0.86
Obese	1.03 (0.89, 1.19)	0.72	0.91 (0.76, 1.09)	0.32
Severely obese	1.12 (0.97, 1.30)	0.12	0.99 (0.83, 1.19)	0.94
Smoking cessation	0.97 (0.92, 1.01)	0.13	–	–
Latest stable blood eosinophil count (absolute count in 10^9^ cells/L)	1.22 (1.03, 1.44)	0.024	–	–
FEV1% predicted	0.99 (0.99, 1.0)	<0.001	0.99 (0.99, 1.0)	<0.001
Exacerbations (number in year prior)	1.02 (1.01, 1.04)	0.033	1.04 (1.02, 1.06)	<0.001
Maintenance therapy during withdrawal period				
No	Reference		Reference	
Yes	0.85 (0.81, 0.89)	<0.001	0.95 (0.88, 1.02)	0.16
Asthma history				
No asthma history	Reference		Reference	
>2 years before COPD diagnosis	1.44 (1.37, 1.52)	<0.001	1.28 (1.18, 1.38)	<0.001
≤2 years before COPD diagnosis or withdrawal date	1.22 (1.15, 1.30)	<0.001	1.12 (1.04, 1.22)	0.004
History of heart disease				
No	Reference		Reference	
Yes	0.91 (0.86, 0.96)	<0.001	0.95 (0.88, 1.03)	0.19
Vaccination history				
No	Reference		–	–
Yes	1.10 (1.01, 1.19)	0.026	–	–
Start of withdrawal coincides with a review				
No	Reference		Reference	
Yes	0.67 (0.62, 0.72)	<0.001	0.86 (0.76, 0.97)	0.01

^a^Age and sex covariates were included in the final model regardless of their threshold for the backward variable selection process.

In the multivariable model, the effect of maintenance therapy alone on ICS-free time was diminished (HR: 0.95; 95% CI: 0.88, 1.02; *P* = 0.16), but with statistical evidence of an interaction with the presence of COPD review period at the start of withdrawal. The interaction between maintenance therapy and COPD review suggested that patients on maintenance therapy who had a COPD review coded coinciding with withdrawal date were 28% less likely (HR: 0.72; 95% CI: 0.61, 0.85; *P* < 0.001) to restart ICS compared with patients with no maintenance therapy that did not have a COPD review at the start of withdrawal (Table 4).

**Table 4:** Modification of the effect of maintenance therapy group on restarting ICS therapy by the COPD review at the date of withdrawal.

	Date of withdrawal coincides with annual/6-month review period			
	No		Yes	
Treatment prescribed	HR^a^ (95% CI)	*P* value	HR^a^ (95% CI)	*P* value
No maintenance	Reference	–	0.86 (0.76, 0.97)	0.01
Maintenance therapy	0.95 (0.88, 1.02)	0.16	0.72 (0.61, 0.85)	<0.001

^a^Hazard ratios correspond to the interaction between subgroups with patients that did not receive a COPD review and maintenance therapy as a reference group.

In addition, COPD review at the start of withdrawal was independently shown to be associated with a longer withdrawal period until ICS restart in both the univariable (HR: 0.67; 95% CI: 0.62, 0.72; *P* < 0.001) and multivariable (HR: 0.86; 95% CI: 0.76, 0.97; *P* = 0.01) models (Table 3).

### Patient outcomes

**Fig. 4:**
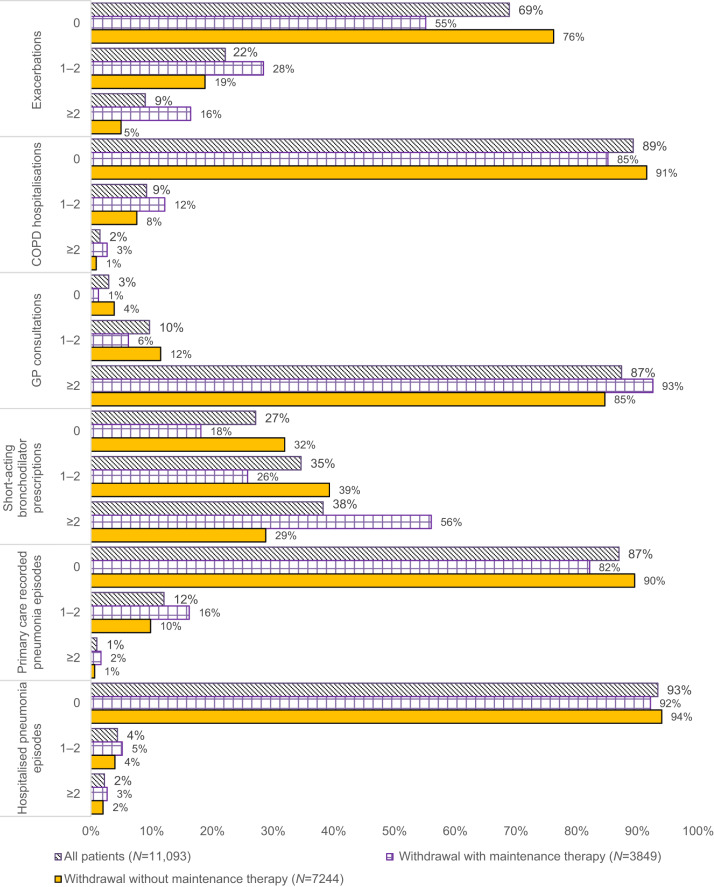
Frequency of clinical outcomes during periods of ICS-free time by the patient group. Clinical outcomes included the number and percentage of patients experiencing exacerbations, hospitalisations, GP consultations, use of short-acting bronchodilator therapies, and pneumonia episodes by those receiving maintenance therapy and those without maintenance therapies.

During the ICS-free period studied, the majority of patients that had withdrawn ICS did not experience any exacerbations (*N* = 7654; 69%), COPD-related hospitalisation (*N* = 9873; 89%), primary care recorded pneumonia episodes (*N* = 9651; 87%), or hospitalised pneumonia episodes (*N* = 10,316; 93%) (Fig. 4). COPD exacerbations were higher in the maintenance therapy group with most of these patients experiencing ≥1 exacerbation event prior to the start of maintenance therapy (*N* = 1693 of 3849 patients, 44%; *P* < 0.001) compared to patients without maintenance therapy (*N* = 2622 of 11,093 patients; 24%). The overall rate of hospitalised pneumonia episodes remained similar but slighter higher in patients receiving maintenance therapy (maintenance therapy: 0.45 per 1000 person-days, *P* = 0.02; no maintenance therapy: 0.41 per 1000 person-days) (Supplementary Table 3).

### Sensitivity analysis

Increasing the minimum time defining the ICS-free period from 6 months to 9 months reduced the number of patients defined as withdrawing ICS therapy from 11,093 to 5544. Of those withdrawing, 2116 patients (38.2%) received maintenance therapy during the withdrawal period and 3428 patients (61.8%) did not receive any maintenance therapy. As in the main analysis, the observed median withdrawal time up to the restart of ICS was longer in patients receiving maintenance therapy than in those that did not (12.6 months; IQR 10.3–17.1) vs. (11.7 months; IQR 10–15.4).

A minority of patients receiving the first maintenance therapy after the first 6 months of ICS-free time (8.9%, *N* = 343 of 3849) were originally categorised in the regression models as having no maintenance therapy to prevent immortal time bias. These patients were reassigned to the maintenance therapy group at the point of their first maintenance therapy prescription to understand the impact of a time-dependent variable in the association between use of maintenance therapy and ICS-free time. The reanalysis of time to ICS restart for these patients showed comparable results to the primary analysis (Supplementary Table 2).

Lastly, the proportion of patients with long-term (12 or more months) ICS termination without an observed restart was calculated for patients receiving maintenance therapy and for patients without maintenance therapy. Patients on maintenance therapy were more likely to remain ICS free for ≥12 months. Approximately 1.4% (*n* = 52 of 3849) patients receiving therapy had an ICS withdrawal >5 years compared with 0.73% (*n* = 53 of 7244) patients without therapy. Although it is not known whether patients restarted ICS after the end of the study period, this subgroup of patients with >5 years of ICS withdrawal may indicate a permanent shift to non-ICS maintenance therapies.

## Discussion

This study describes COPD patients on regular ICS therapy who underwent a period without evidence of ICS prescription of at least 6 months. Of patients who had at least 12 months of ICS use prior to withdrawal, 11,093 (14.4%) withdrew ICS with approximately one-third receiving maintenance therapy at some point during the period following withdrawal. The remaining (65%) of patients that did not receive maintenance therapy in the period following ICS withdrawal may include patients with mild or improving symptoms that needed minimal intervention. In addition, these patients may have been prescribed a short-acting muscarinic antagonist (SAMA) and or short-acting beta-agonist inhaler (SABA) inhaler to control symptoms when needed. In addition, GOLD classification and exacerbation rates between patients receiving maintenance therapy compared to those not receiving maintenance therapy may indicate that this cohort overall had the less severe disease when contrasted to patients receiving inhaled maintenance therapy.

Overall, patients that received maintenance therapy had a longer predicted and observed withdrawal time than those without maintenance therapy and were marginally less likely to restart ICS. Routine COPD review recording coinciding with the start of maintenance therapy and a previous asthma diagnosis were key predictors of time to restarting ICS in the multivariable model. The major concern when withdrawing ICS is the possibility of triggering an exacerbation, but in this population, we found that most patients withdrawing ICS did not experience an exacerbation in the study period.

Despite the higher probability of being prescribed LAMA during withdrawal, patients that received dual therapy (LAMA/LABA or LAMA + LABA) were most likely to benefit with a longer observed ICS-free time compared with patients receiving monotherapies. Although NICE guidelines recommend dual therapy when ICS-based regimens are not needed, the finding in this study provides evidence of varied prescribing behaviour in primary care but further underscores the value of LAMA and LABA dual therapy as a more favourable maintenance treatment^32^.

Patients had a longer period without ICS when routine COPD review was recorded at the start of ICS withdrawal and maintenance therapy was prescribed, compared with patients without a maintenance therapy prescription. Annual patient COPD review with the GP is suggested within the primary care of Quality and Outcomes Framework^33^, but a GP-coded review cannot conclusively indicate that a formal COPD review with the patient took place. Alternatively, it is possible that the codes reflect the GP performing a review of the notes and prescriptions. Despite this possible rationale, COPD review codes were strongly and independently associated with ICS-free time suggesting a possible planned withdrawal and re-evaluation of a current treatment or supportive therapy. Additionally, these codes may also suggest that more reviewing of COPD medication is taking place in primary care.

Although this study was able to demonstrate the varying lengths of ICS-withdrawal period by patient characteristics and exposure to maintenance therapy, the electronic medical records did not include the reason for clinicians to initiate maintenance therapy or ICS re-initiation. As observed, just over one-half of patients receiving maintenance therapy had COPD exacerbations prior to the start of the maintenance therapy. This observation may suggest a confounding by indication bias such that clinicians maybe more likely to prescribe maintenance therapy in those they consider at higher risk of future exacerbations^34^. Consequently, the association between maintenance therapy and clinical outcomes during withdrawal must be interpreted with caution as the directionality of the association cannot be confirmed in this study. The effect of escalating or changing treatment class on withdrawal duration, COPD exacerbations, hospitalisations, and pneumonia episodes was not tested in this study but may provide insight as to how patient outcomes are linked with prescribing behaviours of physicians. Lastly, at baseline, the severity of COPD disease based on FEV1% was unknown in approximately one-half of all patients without maintenance treatment, which may indicate variability of how spirometry results are documented and coded. This variation in practice may result in a possible misdiagnosis of COPD or a possible misrepresentation of airflow limitation severity.

Based on the conclusions of recent meta-analyses, ICS withdrawal does not significantly increase the overall risk of COPD exacerbations; there are differences regarding FEV_1_ decline and quality of life metrics, which are on average below the minimal clinically important difference^35,36^. However, the authors note that current evidence does not evaluate the impact of ICS withdrawal after clustering COPD patients with regard to phenotype characteristics (e.g. frequent exacerbators, emphysema- hyperinflation or COPD with an asthma component), the rate of exacerbations/year (i.e.<1; ≥1 and ≤2; >2), the decline of lung function (rapid decliners vs. slower decliners) or the quality of life^37–39^. Recent studies examining primary and secondary care electronic records in the UK and Germany have also found that withdrawal of ICS is not associated with an increased risk of exacerbations compared to a cohort of COPD patients who continue ICS therapy^40–42^. A specific UK study using a large administrative healthcare database comparing a cohort of patients withdrawing from a triple-therapy ICS-based regimen also suggested no increase in moderate and severe exacerbations due to a higher number of pneumonia consultations recorded in primary care at baseline^42^. The current study adds further information as to the way ICS therapy is withdrawn in a primary care setting. Although a minority of patients received a COPD review when prescriptions for ICS were ceased, these patients were least likely to restart ICS, which suggests that withdrawing ICS may be a part of an ICS-step down plan, an approach that is recommended by The Primary Care Respiratory Society (PCRS)^43^. In this cohort, patients that experienced exacerbations were also more likely to be on maintenance therapy, but it is not possible to draw conclusions from this observation as there may be confounding factors that were not considered, unclassified disease severity groups at baseline, and a likely reverse causality between the need for maintenance therapy and clinical outcomes. For example, in the Copenhagen General Population Study^44^, adherence to maintenance medication for COPD was low, although this increased with the progressive severity of the disease as defined by the GOLD stage.

This study has several strengths to understand the characteristics of patients and associated periods of ICS withdrawal. We utilised data from a large, well-validated primary care database with linkage to secondary care data. The identification of acute exacerbations of COPD from electronic health records is well-validated, and since it is based on data from routine care, the prescription data are likely to be entered correctly as the electronic health record system is required to generate a patient prescription^30,45^. This study provides a good capture of general prescribing behaviour in the COPD patient population as these patients are predominately managed in primary care.

Nevertheless, we cannot know if the prescription was dispensed, nor whether the patient took the medication, and adherence to maintenance medication in COPD is known to be poor^44^. In this study, we were interested in the effects of ICS withdrawal, so we can be relatively confident that the absence of a prescription record indicates that medication has not been supplied. It is likely that these patients were prescribed short-acting bronchodilation therapy or supportive care therapies for the relief of both respiratory and non-respiratory symptoms respectively, suggestive that patients had regular or routine visits to the GP to manage symptoms during the withdrawal period, however; we cannot confirm that all cases of withdrawal are genuinely deliberate attempts to withdraw ICS at the direction of a GP. For some patients, experiencing unusually longer gaps between withdrawal date and re-uptake of ICS therapy could be attributed to failure to refill prescriptions (i.e. patient-driven withdrawal) or stockpiling of previously issued prescriptions of ICS monotherapy or ICS combined therapy with LAMA or LABA. Although previous studies have described how to minimise the risk of steroid withdrawal^22–25^, this study seeks to describe a steroid withdrawal population in practice. The comparison of patients in this study to those that did not withdraw ICS for a minimum of 6 months may provide insight on COPD exacerbations and outcomes, but we cannot guarantee a sufficient patient population of continuous long-term ICS use in addition to the historical 12-month persistent ICS use prior to index date. Patients are more likely to start and stop ICS perhaps due to changing clinical signs or higher exacerbations events compared to those on long-acting therapies only. For this reason, comparison ICS withdrawn patients to ICS persistent users may lead to differential misclassification of outcomes thus weakening the association between clinical outcomes and ICS withdrawal.

This study has also shown that many patients in the withdrawing population had an historic or concurrent asthma diagnosis and the decision to withdraw may be confounded by the coded diagnosis of asthma rather than COPD, with asthma diagnosis possibly over-recorded in individuals with COPD^46^. This may explain why concomitant asthma increased the risk of restarting ICS, as these patients were being managed more as asthma patients than COPD, although severity criteria specifically for asthma (e.g. Asthma Control Test score) were not captured in this study. Historical asthma diagnosis was also more prevalent in patients who did not receive maintenance therapy, which may suggest that these patients have stepped off ICS due to improvement of asthmatic symptoms.

Results from the IMPACT^39^ and SUNSET^47^ clinical trials suggest that the risk of exacerbations following ICS withdrawal, although small, primarily occurs in the first 28 days^37^ and the step down from LAMA/LABA in combination with ICS-based regimen to LAMA/LABA dual maintenance therapy show no significant difference in COPD exacerbations, especially in patients with blood eosinophils <0.3 × 10^9^ cells/L^45^. Although the IMPACT trial has highlighted that sudden step down from triple therapy to dual maintenance therapy can introduce exacerbations, it may be due to the fact these patients were frequent exacerbators and characterised with asthmatic features who may have otherwise benefited from continued ICS treatment. Overall, these findings suggest that withdrawal must be carefully investigated on a case-by-case basis based on multiple patient-based factors. Our definition of withdrawal included a minimum 6-month ICS-free period and therefore could not specifically identify the immediate increased risks of ICS- withdrawal within this study, and therefore direct comparison with IMPACT and SUNSET is not possible. Our data provided insight into characteristics and longer-term outcomes of patients that were not restarted on ICS due to short-term reactions to withdrawal.

Whether withdrawal of ICS occurs in a planned or unplanned way, the majority of COPD patients withdrawing ICS did not experience exacerbations and COPD-related hospitalisations. When ICS-withdrawal occurs in a planned way with a COPD review taking place and maintenance therapy prescribed, the ICS-free period is longer compared to patients without a prescribed maintenance therapy or a recorded COPD review. This may result in the reduction of side effects and enable more cost-effective prescribing. This study provides evidence that ICS withdrawal results in minimal exacerbations of COPD, thus supporting the guidance on recent ERS ICS withdrawal^21^. Withdrawal success can be optimised by carefully planning COPD reviews whilst optimising the use of available maintenance therapies.

## Supplementary information

Supplementary Info

REPORTING SUMMARY

## Data Availability

Datasets are available on request from the CPRD. Their provision requires the purchase of a license, and our license does not permit us to make them publicly available to all. We used data from the version collected in January 2019 and have clearly specified the data selected in our Methods section. To allow identical data to be obtained by others, via the purchase of a license, we will provide the code lists on request. Licences are available from the CPRD (http://www.cprd.com): The Clinical Practice Research Datalink Group, The Medicines and Healthcare products Regulatory Agency, 5th Floor, 151 Buckingham Palace Road, Victoria, London SW1 W 9SZ.

## Code availability

Statistical programming code that is customised for this manuscript analysis is completed in STATA version 15 and can be accessed upon request.
