# Supplementary material for: A descriptive cohort study of withdrawal from inhaled corticosteroids in COPD patients
Source: NPJ Prim Care Respir Med. 2022 Jul 20;32:25. doi: 10.1038/s41533-022-00288-6 (PMC9300648; doi:10.1038/s41533-022-00288-6)
Supplement: Supplementary file 1 — Supplementary Info [file 41533_2022_288_MOESM1_ESM.docx]

Supplementary Figure 1. Mean withdrawal period by year of ICS withdrawal and patient group

Error bars reflect 95% confidence interval

Supplementary Table 1. Loss-to follow up reasons in patients with no subsequent prescription of ICS

|  |  |  |
| --- | --- | --- |
| **Reason** | **N** | **%** |
| Total patients in study | 11,093 | 100 |
|  |  |  |
| No longer of research quality | 1,567 | 14.1 |
| Transferred out of current GP practice | 497 | 4.5 |
| Death | 446 | 4 |
| Total patients reaching end of study period | 739 | 6.7 |
| Total patients lost to follow-up | 3,249 | 29.3 |

Number and percentage of patients attributed to a reason for loss to follow-up during the study period

Supplementary Table 2. Multivariable model of time without ICS based on patient characteristics (Hazard ratios >1 present increased risk of restarting ICS or shorter-ICS free time) for patients re-assigned to maintenance therapy patient group

|  |  | **Multivariable Analysis**  **(N=6,009)** | | | |
| --- | --- | --- | --- | --- | --- |
| **Characteristic** |  | **HR (95% CI)** | **P-value** | | |
| Age |  |  |  | | |
| 35-44 |  | Ref |  | | |
| 45-54 |  | 0.95 (0.73,1.24) | 0.73 | | |
| 55-64 |  | 0.97 (0.76,1.25) | 0.84 | | |
| 65-74 |  | 0.96 (0.75,1.23) | 0.76 | | |
| 75-80 |  | 0.97 (0.75,1.24) | 0.78 | | |
| >80 |  | 0.93 (0.72,1.20) | 0.60 | | |
| Sex |  |  |  | | |
| Female |  | Ref |  | | |
| Male |  | 0.95 (0.89,1.01) | 0.12 | | |
| Body mass index (BMI) kg/m^2^ |  |  |  | | |
| Normal |  | Ref |  | | |
| Underweight |  | 0.98 (0.79,1.21) | 0.83 | | |
| Overweight |  | 1.01 (0.85,1.21) | 0.88 | | |
| Obese |  | 0.91 (0.76,1.09) | 0.31 | | |
| Severely Obese |  | 0.99 (0.83,1.19) | 0.94 | | |
| FEV1% Predicted |  | 0.99 (0.99,1.0) | <0.001 | | |
| Exacerbations (number in year prior) |  | 1.04 (1.02,1.05) | <0.001 | | |
| Maintenance Therapy during Withdrawal period |  |  |  | | |
| No |  | Ref |  | | |
| Yes |  | 0.97 (0.90,1.04) | 0.37 | | |
| Asthma History |  |  |  | | |
| No Asthma history |  | Ref |  | | |
| >2 years before COPD diagnosis |  | 1.28 (1.18,1.38) | <0.001 | | |
| ≤ 2 years before COPD diagnosis or withdrawal date |  | 1.13 (1.04,1.22) | 0.003 | | |
| History of Heart Disease |  |  |  | | |
| No |  | Ref |  | | |
| Yes |  | 0.95 (0.88,1.03) | 0.19 | | |
| Start of withdrawal coincides with a review |  |  |  | | |
| No |  | Ref |  | | |
| Yes |  | 0.84 (0.74,0.95) | 0.007 | | |
|  |  | | |  |  |

Multivariable analysis was conducted through a complete case count using a backward variable selection process with p=0.2 as a threshold for variable selection. Age and sex covariates were included in final model regardless of threshold.

Supplementary Table 3. Rate of clinical outcomes by year of ICS withdrawal start and patient group

|  | **Maintenance Therapy** | | | | | |  |  | **No maintenance therapy** | | | | | |  |  |
| --- | --- | --- | --- | --- | --- | --- | --- | --- | --- | --- | --- | --- | --- | --- | --- | --- |
|  | **2012** | **2013** | **2014** | **2015** | **2016** | **2017** | **Overall Rate**  **per 1,000 days** |  | **2012** | **2013** | **2014** | **2015** | **2016** | **2017** | **Overall Rate**  **per 1,000 days** | **P-value** |
| **Total patients (N,%)** | 1,090 | 1037 | 712 | 477 | 370 | 163 |  |  | 2357 | 2035 | 1334 | 860 | 463 | 195 |  |  |
| **Mean withdrawal months (SD)** | 15.7 (14.3) | 15.8 (13.6) | 13.3 (10) | 13.8 (9.1) | 13 (5.7) | 8.9 (2) |  |  | 12.8 (11.1) | 12.4(9.9) | 12.1 (8.9) | 11.9 (7.6) | 11.2 (5.3) | 8.2 (1.8) |  |  |
| **Total withdrawal person days** | 514,356 | 491,071 | 284,522 | 197,373 | 144,345 | 43,303 |  |  | 904,199 | 759,775 | 484,124 | 306,775 | 155,963 | 48,009 |  |  |
| **Exacerbations** |  |  |  |  |  |  |  |  |  |  |  |  |  |  |  |  |
| Total events | 1,448 | 1,315 | 820 | 629 | 464 | 133 | 4,809 |  | 1,173 | 922 | 549 | 468 | 228 | 54 | 3,394 | <0.001 |
| Rate per 1,000 days | 2.8 | 2.7 | 2.9 | 3.2 | 3.2 | 3.1 | 2.9 |  | 1.3 | 1.2 | 1.1 | 1.5 | 1.5 | 1.1 | 1.3 |  |
| **COPD Hospitalizations** |  |  |  |  |  |  |  |  |  |  |  |  |  |  |  |  |
| Total events | 299 | 272 | 156 | 146 | 110 | 33 | 1,016 |  | 290 | 239 | 177 | 134 | 80 | 20 | 940 | <0.001 |
| Rate per 1,000 days | 0.58 | 0.55 | 0.55 | 0.74 | 0.76 | 0.76 | 0.61 |  | 0.32 | 0.31 | 0.37 | 0.44 | 0.51 | 0.42 | 0.35 |  |
| **GP consultations** |  |  |  |  |  |  |  |  |  |  |  |  |  |  |  |  |
| Total events | 21,397 | 21,383 | 11,901 | 8,252 | 6,511 | 1,823 | 71,267 |  | 33,803 | 28,490 | 17,149 | 11,854 | 5741 | 1,774 | 98,811 | <0.001 |
| Rate per 1,000 days | 41.6 | 43.5 | 41.8 | 41.8 | 45.1 | 42.1 | 42.5 |  | 37.4 | 37.5 | 35.4 | 38.6 | 36.8 | 37 | 37.2 |  |
| **Short-acting bronchodilator prescriptions (SABA)** |  |  |  |  |  |  |  |  |  |  |  |  |  |  |  |  |
| Total events | 6,760 | 6,918 | 4,143 | 3010 | 2,224 | 683 | 23,738 |  | 6,823 | 5,391 | 3,436 | 2577 | 1084 | 366 | 19,677 | <0.001 |
| Rate per 1,000 days | 13.1 | 14.1 | 14.6 | 15.3 | 15.4 | 15.8 | 14.2 |  | 7.5 | 7.1 | 7.1 | 8.4 | 7 | 7.6 | 7.4 |  |
| **Primary care recorded pneumonia** |  |  |  |  |  |  |  |  |  |  |  |  |  |  |  |  |
| Total events | 312 | 307 | 152 | 113 | 87 | 22 | 993 |  | 345 | 301 | 170 | 115 | 79 | 7 | 1,017 | <0.001 |
| Rate per 1,000 days | 0.61 | 0.63 | 0.53 | 0.57 | 0.6 | 0.51 | 0.59 |  | 0.38 | 0.4 | 0.35 | 0.37 | 0.51 | 0.15 | 0.38 |  |
| **Hospitalized pneumonia** |  |  |  |  |  |  |  |  |  |  |  |  |  |  |  |  |
| Total events | 262 | 183 | 124 | 92 | 86 | 13 | 760 |  | 277 | 310 | 200 | 141 | 129 | 20 | 1,077 | 0.02 |
| Rate per 1,000 days | 0.51 | 0.37 | 0.44 | 0.47 | 0.6 | 0.3 | 0.45 |  | 0.31 | 0.41 | 0.41 | 0.46 | 0.83 | 0.42 | 0.41 |  |

P-value indicates statistical difference between overall rate comparing patients receiving maintenance therapy to patients not receiving maintenance therapy. Overall rates are defined as the crude event rate during follow-up.
