# Supplementary material for: A descriptive cohort study of withdrawal from inhaled corticosteroids in COPD patients
Source: NPJ Prim Care Respir Med. 2022 Jul 20;32:25. doi: 10.1038/s41533-022-00288-6 (PMC9300648; doi:10.1038/s41533-022-00288-6)
Supplement: Supplementary file 2 — REPORTING SUMMARY [file 41533_2022_288_MOESM2_ESM.pdf]

nature portfolio

Corresponding author(s): Smit Patel

Last updated by author(s): May 9, 2022

Reporting Summary

Nature Portfolio wishes to improve the reproducibility of the work that we publish. This form provides structure for consistency and transparency in reporting. For further information on Nature Portfolio policies, see our Editorial Policies and the Editorial Policy Checklist.

Statistics

For all statistical analyses, confirm that the following items are present in the figure legend, table legend, main text, or Methods section.

n/a	Confirmed	
☐	☒	The exact sample size (n) for each experimental group/condition, given as a discrete number and unit of measurement
☐	☒	A statement on whether measurements were taken from distinct samples or whether the same sample was measured repeatedly
☐	☒	The statistical test(s) used AND whether they are one- or two-sided<br>*Only common tests should be described solely by name; describe more complex techniques in the Methods section.*
☐	☒	A description of all covariates tested
☐	☒	A description of any assumptions or corrections, such as tests of normality and adjustment for multiple comparisons
☐	☒	A full description of the statistical parameters including central tendency (e.g. means) or other basic estimates (e.g. regression coefficient) AND variation (e.g. standard deviation) or associated estimates of uncertainty (e.g. confidence intervals)
☐	☒	For null hypothesis testing, the test statistic (e.g. F, t, r) with confidence intervals, effect sizes, degrees of freedom and P value noted<br>*Give P values as exact values whenever suitable.*
☒	☐	For Bayesian analysis, information on the choice of priors and Markov chain Monte Carlo settings
☒	☐	For hierarchical and complex designs, identification of the appropriate level for tests and full reporting of outcomes
☒	☐	Estimates of effect sizes (e.g. Cohen's d, Pearson's r), indicating how they were calculated

Our web collection on statistics for biologists contains articles on many of the points above.

Software and code

Policy information about availability of computer code

Data collection	No software was used for data collection
Data analysis	STATA version 15 and SAS version 9.4 was used for data clean up, analysis, and reporting

For manuscripts utilizing custom algorithms or software that are central to the research but not yet described in published literature, software must be made available to editors and reviewers. We strongly encourage code deposition in a community repository (e.g. GitHub). See the Nature Portfolio guidelines for submitting code & software for further information.

Data

Policy information about availability of data

All manuscripts must include a data availability statement. This statement should provide the following information, where applicable:

- Accession codes, unique identifiers, or web links for publicly available datasets
- A description of any restrictions on data availability
- For clinical datasets or third party data, please ensure that the statement adheres to our policy

Datasets are available on request from the CPRD. Their provision requires the purchase of a license, and our license does not permit us to make them publicly available to all. We used data from the version collected in January 2019 and have clearly specified the data selected in our Methods section. To allow identical data to be obtained by others, via the purchase of a license, we will provide the code lists on request. Licences are available from the CPRD (http://www.cprd.com): The Clinical Practice Research Datalink Group, The Medicines and Healthcare products Regulatory Agency, 5th Floor, 151 Buckingham Palace Road, Victoria, London SW1 W 9SZ.

Field-specific reporting

Please select the one below that is the best fit for your research. If you are not sure, read the appropriate sections before making your selection.

☐ Life sciences ☒ Behavioural & social sciences ☐ Ecological, evolutionary & environmental sciences

For a reference copy of the document with all sections, see nature.com/documents/nr-reporting-summary-flat.pdf

Behavioural & social sciences study design

All studies must disclose on these points even when the disclosure is negative.

Study description	This is a retrospective cohort study based on patient electronic medical records in primary and secondary care
Research sample	This study includes COPD patients registered to primary care general practices in the UK between January 2012 and December 2017. Patients had to be at least 35 years of age at diagnosis with good research quality data in CPRD and atleast 12 months with inhaled corticosteroid (ICS) use prior to ICS withdrawal. Additionally, patients need to be linked to secondary care electronic records, with at least 1 prescription of ICS during the study period.
Sampling strategy	Patients were sampled based on specific medical diagnosis codes indicating a diagnosis of COPD registered in the UK primary care electronic medical records database.
Data collection	Patient data is electronically included in a primary care database across the UK through the General Practioner software. Patient data is recruited into cohorts based on data extraction of specified inclusion criteria electronically.
Timing	We will recruits patient data from 01 January 2012 to 31st December 2017 as the study period
Data exclusions	11,614 patients out of 22,707 were excluded from the analysis as these patients had at least 1 withdrawal of less than 6 months from ICS within the study period
Non-participation	Non-participation rates were not identified in this study due the nature of the study design and data source. Patient data was identified solely through diagnosis codes from primary care electronic records.
Randomization	Patients were not randomized due to the descriptive nature of the study.

Reporting for specific materials, systems and methods

We require information from authors about some types of materials, experimental systems and methods used in many studies. Here, indicate whether each material, system or method listed is relevant to your study. If you are not sure if a list item applies to your research, read the appropriate section before selecting a response.

Materials & experimental systems

n/a	Involved in the study	
☒	☐	Antibodies
☒	☐	Eukaryotic cell lines
☒	☐	Palaeontology and archaeology
☒	☐	Animals and other organisms
☐	☒	Human research participants
☒	☐	Clinical data
☒	☐	Dual use research of concern

Methods

n/a	Involved in the study	
☒	☐	ChIP-seq
☒	☐	Flow cytometry
☒	☐	MRI-based neuroimaging

Human research participants

Policy information about studies involving human research participants

Population characteristics	Patients in this study were required to be atleast 35 years of age, registered in a general practice with data linked to secondary care health records. Additionally, patients must have continuous inhaled corticosteroid use for 1 year prior to withdrawal of inhaled corticosteroid.
Recruitment	Cohort recruitment included retrospective data extraction of patient electronic medical records in the clinical practice research data link (CPRD). Patients were identified by diagnosis codes read in primary care databases and further filtered according to patient eligibility and inclusion criteria. As patients are linked to secondary care (hospitalisation records) data as part of eligibility, these patients may tend to experience more symptoms, co-diagnosis such as asthma, or severity compared to those patients managed solely by primary care services. Although we do not expect a majority of patients to have asthma

	co-diagnosis, the presence of such similar conditions may potentially overestimate the number of ICS prescriptions and how likely a GP will consider an ICS step-down approach.
Ethics oversight	The use for CPRD data for research purposes received ethical approval by an Independent Scientific Advisory Committee for Medicines and Health care products Regulatory Agency (ISAC -MHRA) - ISAC number 17195RA. As this was a non-interventional study using anaonymized data, no patient consent was necessary.

Note that full information on the approval of the study protocol must also be provided in the manuscript.
